# Xin-Ji-Er-Kang Alleviates Myocardial Infarction-Induced Cardiovascular Remodeling in Rats by Inhibiting Endothelial Dysfunction

**DOI:** 10.1155/2019/4794082

**Published:** 2019-06-25

**Authors:** Pan Cheng, Feng-zhen Lian, Xiao-yun Wang, Guo-wei Cai, Guang-yao Huang, Mei-ling Chen, Ai-zong Shen, Shan Gao

**Affiliations:** ^1^Department of Pharmacology, Basic Medical College, Anhui Medical University, Hefei 230032, China; ^2^Cancer Hospital, Chinese Academy of Sciences, Hefei 230032, China; ^3^The First Affiliated Hospital of USTC, Division of Life Sciences and Medicine, University of Science and Technology of China, Hefei, Anhui 230001, China

## Abstract

The present study was designed to elucidate the beneficial effects of XJEK on myocardial infarction (MI) in rats, especially through the amelioration of endothelial dysfunction (ED). 136 Sprague-Dawley rats were randomized into 13 groups: control group for 0wk (*n* = 8); sham groups for 2, 4, and 6 weeks (wk); MI groups for 2, 4, and 6 wk; MI+XJEK groups for 2, 4, and 6w k; MI+Fosinopril groups for 2, 4, and 6 wk (*n* = 8~10). In addition, 8 rats were treated for Evans blue staining and Tetrazolium chloride (TTC) staining to determine the infarct size. Cardiac function, ECG, and cardiac morphological changes were examined. Colorimetric analysis was employed to detect nitric oxide (NO), and enzyme-linked immunosorbent assay (ELISA) was applied to determine N-terminal probrain natriuretic peptide (NT-ProBNP), endothelin-1 (ET-1), angiotensin II (Ang II), asymmetric dimethylarginine (ADMA), tetrahydrobiopterin (BH_4_), and endothelial NO synthase (eNOS) content. The total eNOS and eNOS dimer/(dimer+monomer) ratios in cardiac tissues were detected by Western blot. We found that administration of XJEK markedly ameliorated cardiovascular remodeling (CR), which was manifested by decreased HW/BW ratio, CSA, and less collagen deposition after MI. XJEK administration also improved cardiac function by significant inhibition of the increased hemodynamic parameters in the early stage and by suppression of the decreased hemodynamic parameters later on. XJEK also continuously suppressed the increased NT-ProBNP content in the serum of MI rats. XJEK improved ED with stimulated eNOS activities, as well as upregulated NO levels, BH_4_ content, and eNOS dimer/(dimer+monomer) ratio in the cardiac tissues. XJEK downregulated ET-1, Ang II, and ADMA content obviously compared to sham group. In conclusion, XJEK may exert the protective effects on MI rats and could continuously ameliorate ED and reverse CR with the progression of MI over time.

## 1. Introduction

Globally, myocardial infarction (MI) has become the leading contributor to the burden of diseases associated with increased risk of heart failure and mortality [1], in spite of the tremendous research efforts over the past years [2]. MI is defined as a pathological event involving ventricular remodeling and myocardial cell necrosis due to significant and sustained ischemia [3]. Being considered as an early response to preserve cardiac function, cardiac hypertrophy can lead to heart failure although the mechanisms involved in the transition are poorly understood [4].

Endothelial dysfunction (ED), characterized by decreased nitric oxide (NO) bioavailability, appears to have a deleterious effect during the long-term process of remodeling [5]. Under physiological conditions, functional endothelial NO synthase (eNOS), together with the redox-sensitive cofactor tetrahydrobiopterin (BH_4_), works as a dimeric protein to produce NO, and the eNOS-derived NO serves to promote vascular homeostasis and might affect cardiac myocyte function [6]. However, ventricular remodeling process after MI leads to BH_4_ oxidation, resulting in the uncoupled eNOS-derived superoxide generation, which further augments the remodeling process and deteriorates cardiac function [7]. In addition, endothelin (ET-1), a endothelial-derived vasoconstrictor peptide, maintains the vascular tone in healthy humans. However, its expression and endothelin receptor A (ET_A_) levels are upregulated in various cardiovascular disorders like spontaneous hypertension [8], myocardial infarction [9], and atherosclerosis [10]. Evidently, the renin-angiotensin system (RAS) is integrally involved in the genesis and progression of various cardiovascular diseases. When RAS is activated, angiotensin II (Ang II) becomes elevated, simultaneously impairing eNOS activity and increasing ET-1 levels [11]. Zhou et al. have reported that not only NO bioavailability but also the imbalance between eNOS-derived NO and ET-1 contributes to ED, ultimately aggravating the MI [12].

Xin-Ji-Er-Kang (XJEK) is a traditional Chinese herbal formula made of fourteen herbal medicines, such as* Astragalus mongholicus Bunge, Ophiopogon japonicus (Thunb.) KerGawl, Polygonatum odoratum (Mill.) Druce*,* Panax ginseng, C.A. Mey., *and some other ingredients as well. Numerous clinical and basic researches have revealed the protective effects of XJEK on viral myocarditis [13], MI induced cardiovascular injury [14], and 2-kidney 1-clip (2K1C) induced hypertension [15, 16]. Our previous studies have shown that the doses of XJEK from 4 to 12 g/kg/day, especially 8 g/kg/day treatment for 4 weeks, may protect against inflammation, oxidative stress, and MI induced ED in mice [17]. However, it is as yet unclear whether XJEK continues to play a role in MI rats over time or not. This study therefore sets out to assess the effects of XJEK (6.2 g/kg/day, calculated from dose of mice) on cardiac function abnormalities, cardiovascular remodeling, and ED over time and attempts to explore the potential mechanisms focusing on ED.

## 2. Materials and Methods

### 2.1. Animals and Chemicals

All procedures were approved by the Institutional Animal Care and Use Committee of Anhui Medical University. A total of 136 male Sprague-Dawley rats (220–250 g) were obtained from Shanghai Slac Laboratory Animal Corp. Ltd. (Certificate No.SCXK (HU) 2012-0002) and ventilated with room air. These animals were randomized into the following groups: control group for 0wk (*n* = 8); sham groups for 2, 4, and 6 weeks (wk); MI groups for 2, 4, and 6 wk; MI+XJEK groups for 2, 4, and 6 wk; MI+Fosinopril groups for 2, 4, and 6wk (*n* = 8~10). Another eight rats were treated for Evans blue staining and Tetrazolium chloride (TTC) staining. Animals in XJEK treatment groups received an intragastric gavage with XJEK at 6.2 g/kg/d (calculated from dose of mice); Fosinopril treatment groups were administered 1.5mg/kg/d by intragastric gavage, while those in sham and MI groups were dealt with distilled water. Fosinopril is an angiotensin converting enzyme inhibitor that effectively reduces vascular resistance and improves cardiac output. XJEK was acquired from the Hefei Seven Star Medical Science and Technology Company and Fosinopril was obtained from Bristol-Myers Squibb (Shanghai, China, AAM6233).

### 2.2. Establishment of MI Model and Measurement of Infarct Area at Risk

The MI model was induced by ligation of left anterior descending coronary artery (LAD) and animals undergoing a sham operation were similarly treated, except that the suture around the coronary artery only passed through the muscle without being tied as described previously [12]. Briefly, male rats were anesthetized with sodium pentobarbital 1% (50 mg/kg,* i.p.*) and ventilated with positive pressure via a tube inserted into the trachea and connected to a small animal respirator (BL420S, Chengdu Techman Software Co., Ltd, China). When the adequacy of anesthesia was monitored by observation of slow breathing, loss of muscular tone, and no response to surgical manipulation, a left thoracotomy was performed via the third intercostals space, and the left anterior descending coronary artery was ligated using a 5-0 silk suture. Then, the thoracotomy site was closed. The successful MI model was confirmed not only by real-time ECG monitoring, i.e., a ST segment elevation, but also by visual inspection of LV color alteration. After surgery all animals were injected with cefoxitin sodium (200 mg/kg/day) for three consecutive days.

Twenty-four hours after ligation of LAD, heart sections of eight rats were stained with Evans blue/TTC to determine the infarct size as previously described [18]. Evans blue stained areas indicated nonischemia area. Blue, white, and red parts in the heart represented normal myocardium, infarct area, and ischemic area, respectively. White plus red part indicated the area at risk. Photos were captured using a digital camera, and then the relative infarct size could be analyzed with Image J (1.61).

### 2.3. Measurement of ECG

During the experimental process, the BL-420 biological function experiment system was used in order to monitor and record the electrocardiogram (ECG) of standard limb lead II as described previously [19]. The height and width of P, T, S wave, QT interval, and P-R interval of baseline, 1 min after MI and 2, 4, and 6 wks after MI, were measured, respectively, using the image analysis software. The ECG changes of each time point were compared among groups.

### 2.4. Haemodynamic Parameters

At the end of 0, 2, 4, and 6 wks after MI, the haemodynamic parameters were assessed. Animals were anesthetized with sodium pentobarbital 1% (50 mg/kg,* i.p.*), respectively; then the right carotid artery was cannulated with a polyethylene catheter connected to a Statham transducer, and the mean carotid artery pressure was measured. After advancing the catheter inserted into the left ventricle along the right coronary artery, the signals were noted down on a four-channel acquisition system (BL420S; Chengdu Techman Software Co. Ltd). The admittance catheters need to be soaked for a while in the heparin saline before its insertion into the common carotid artery to prevent clotting. The left ventricular systolic pressure (LVSP), left ventricular end-diastolic pressure (LVEDP), and rate of rise of left ventricular pressure (±dp/dt_max_) were recorded respectively.

### 2.5. Collection of Serum, Thoracic Aorta and Cardiac Tissues

After haemodynamic index detection, blood samples were collected for 10ml from the heart into tubes pretreated with heparin and centrifuged 3500r/min for 10 minutes at the temperature of 4°C; then the supernatant was in storage at −80°C for future analysis as previously described [20]. Cervical exsanguinations were performed on rats, and the heart weight index (HW/BW) was calculated by dividing HW by BW after the harvest and weighing of hearts. Lastly, being separated into several parts, heart samples were fixed in 10% neutral buffer formalin for morphological and immunofluorescence detection or stored in liquid nitrogen for further analyses. Thoracic aortas were also evacuated from rats and then cleaned and maintained in neutral 10% buffered formalin for further morphological detection.

### 2.6. Measurement of NO and Enzyme-Linked Immunosorbent Assay (ELISA)

NO levels were assessed using NO detection Kit (Nanjing Jiancheng Bioengineering Institute, Nanjing, China) following the manufacturer's instructions. ET-1, Ang II, BH_4_, ET_A_, N-terminal probrain natriuretic peptide (NT-ProBNP), asymmetric dimethylarginine (ADMA), and eNOS content in serum or cardiac tissues were assessed by ELISA Kit (Jiangsu Zeyu Biological Technology Co., Ltd, Yancheng, China.) according to the manufacturer's instructions.

### 2.7. Histological and Morphological Analyses of Heart and Thoracic Aorta

Cardiac tissues and thoracic aorta embedded in paraffin were cut into 5*μ*m thick slices and then dewaxed and performed with haematoxylin and eosin (H&E) or Van Gieson (VG) staining. Afterwards, the myocyte cross-sectional area (CSA), thoracic aorta CSA, total aorta (TAA), area of lumen (LA), aorta radius (AR), luminal radius (LR), and media thickness (MT) of aorta were estimated using Image J (1.61) in digitalized microscopic images. Collagen deposition in cardiac tissues was preliminarily evaluated by VG staining. Stained cardiac tissues were photographed at 200× by microscope. Collagen deposition was then assessed by the mean optical density of red area. Perivascular collagen area (PVCA) and collagen volume fraction (CVF) were observed under optical microscope as previously described by Ahmed et al. [21] and Bai et al. [22].

### 2.8. Immunofluorescence

The methods of measuring ET_A_ by Immunofluorescence had been described previously [23]. Briefly, the hearts were fixed with 10% neutral buffer formalin, embedded in paraffin and sectioned into 5*μ*m thick slices. After deparaffinization and antigen activation, sections were incubated with 1:200 primary ET_A_ monoclonal antibody (Beijing Biosynthesis biotechnology Co., Ltd.) dilution overnight at 4°C. Goat anti-rabbit IgG antibody labeled with FITC was used as secondary antibodies. Next, sections were sufficiently rinsed in PBS and then incubated with secondary antibody for 60 min. After rinsing in PBS and mounting on slides with mounting medium with DAPI (Sigma-Aldrich), the fluorescent images were captured by inverted fluorescence microscope (Olympus IX71, Japan).

### 2.9. Western Blotting Analysis

Proteins were extracted from frozen tissue of the cardiac tissues in control (0 wk), sham, MI, XJEK and Fosinopril (0, 2, 4, and 6 wk) groups, respectively. Protein concentration was determined by BCA Protein Assay Kit (Beyotime). LV myocardial homogenates were subjected to sodium dodecyl sulphate-polyacrylamide gel electrophoresis (SDS-PAGE) on 8–10% polyacrylamide gel, and proteins were electroblotted on the PVDF membranes (Immobilon-P; Millipore, Bedford, MA, USA). After blocking in 5% nonfat milk solution at 37°C for 1 h, the membranes were soaked with the following primary antibodies in TBS-T solutions overnight at 4°C: rabbit anti-eNOS antibody (1:1000, Abcam, USA.), rabbit anti-GAPDH antibody (1:1000, Affinity, USA), or rabbit anti-ET_A_ (Biosynthesis biotechnology, Beijing.). After incubation with secondary antibodies (goat anti-rabbit IgG, 1:5000; Affinity, USA), the gray value of each band was detected using the super signal enhanced chemiluminescence (ECL; Amersham Biosciences, Little Chalfont, UK) detection system. The relative band intensity was determined using GAPDH as a loading control by ImageJ software. To evaluate the eNOS dimer/ (dimer+monomer) ratio, SDS-resistant eNOS dimers were detected using nondenaturing conditions and low-temperature SDS-PAGE as previously reported and with minor modification [24].

### 2.10. Statistical Analysis

Results were expressed as mean±SEM. Statistical analysis was performed with two-tailed Student's t-test and one-way analysis of variance (ANOVA). Difference was taken statistically significant at* P*< 0.05.

## 3. Results 

### 3.1. Survival Rate and Infarct Area 24h after Myocardial Infarction

**Figure 1:**
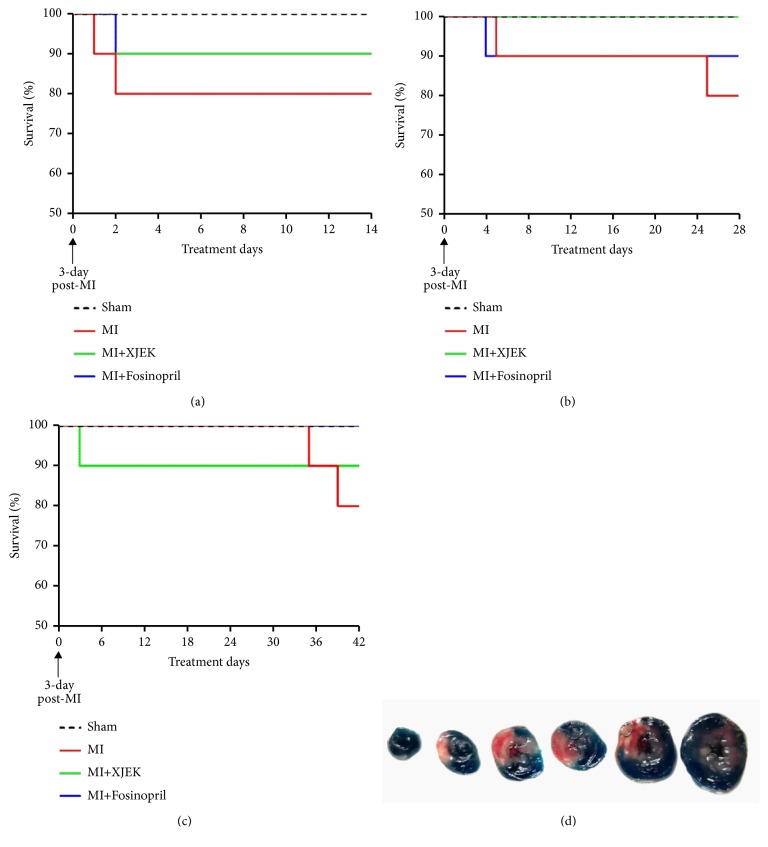
*Survival rate and infarct area 24h after myocardial infarction*. (a) The survival curve of the 2-week group. (b) The survival curve of the 4-week group. (c) The survival curve of the 6-week group. (d) Representative TTC staining. Infarct size was calculated and quantified by Image J (1.61) (*n*=8).

The survival curve was recorded during the drug treatment period of 2, 4, and 6 wk with XJEK. The data indicated that both XJEK and Fosinopril reduced the mortality of rats with myocardial infarction to a certain extent, but it was not statistically significant (Figures 1(a), 1(b), and 1(c)). As shown in Figure 1(a), white parts in the heart indicated the infarct area, while red represented for ischemic tissue and blue indicated normal myocardium. White plus red part was the area at risk. The result showed that the percentage of infarct area was 37.43±3.21% at 24 h after MI.

### 3.2. Effects of XJEK on ECG Remodeling in MI Rats

**Figure 2:**
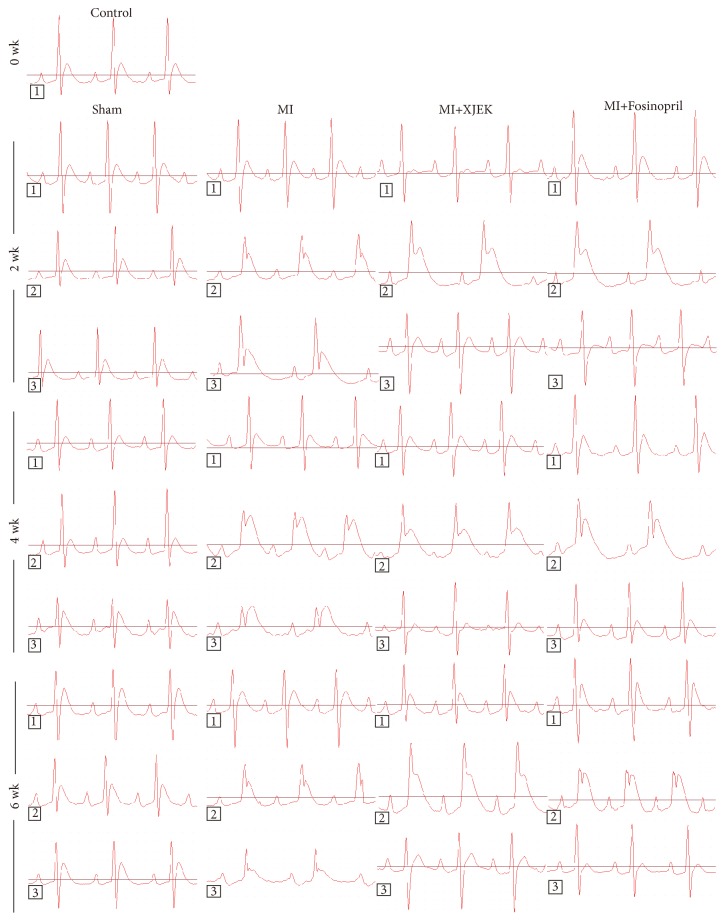
*Effects of XJEK on ECG remodeling in MI rats*. (1) Representative figures of each group on basic (animals were anesthetized but did not receive mechanically ventilated); (2) Representative figures of each group on ischemia 1min (animals were anesthetized and receive mechanically ventilated); (3) Representative figures of each group on ischemia 2, 4, and 6 wk (animals were anesthetized but did not receive mechanically ventilated).

ECG was monitored during the surgical procedures (Figure 2). The T wave in MI rats group rose obviously after coronary artery ligation compared with that in sham groups. Moreover, the width of P, T, and Q-T interval and the P-R interval increased markedly compared to those in sham groups throughout the 6-week experimental period (2, 4, and 6 wk,* P*<0.05 or* P*<0.01). However, XJEK (6.2 g/kg) and Fosinopril (1.5 mg/kg) treatment significantly reduced the height and the width of P and T wave and decreased markedly the time of Q-T interval and P-R interval compared with those in MI group (2, 4, and 6 wk,* P*<0.05 or* P*<0.01; Table 1).

### 3.3. Effects of XJEK on Cardiac Function Injury in MI Rats

We investigated the cardiac function 2, 4, and 6 wk after operation, respectively. There were significant differences in the cardiac function parameters, i.e., HR, ASBP, LVSP, and ±dp/dtmax between sham group and MI group. Compared with sham group, HR was upregulated slightly and there was a significant increase in ASBP, LVSP, and ±dp/dtmax of MI groups for 2 wk and 4 wk (2 wk and 4 wk,* P*<0.05 or* P*<0.01). In MI group for 6 wk; however, LVSP, LVEDP, +dp/dtmax, and -dp/dtmax were greatly downregulated compared with those of sham group (Table 2). These changes could be blocked by treatment with XJEK for 2, 4, and 6 wk, and so could the treatment of Fosinopril.

### 3.4. Effects of XJEK on NT-ProBNP Content in MI Rats

**Figure 3:**
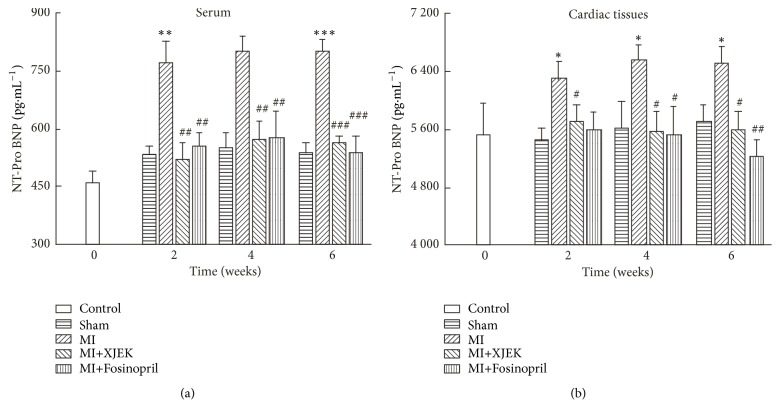
*Effects of XJEK on NT-ProBNP content in MI rats*. (a) NT-ProBNP content in serum; (b) NT-ProBNP content in cardiac tissues. Data are represented as mean ±SEM (*n*=8~10). ^*∗*^*P*<0.05, ^*∗∗*^*P*<0.01, and ^*∗∗∗*^*P*<0.001 versus sham group; ^*#*^*P*<0.05, ^*##*^*P*<0.01, and ^*###*^*P*<0.001 versus MI group.

Serum NT-ProBNP content was significantly higher in the MI rats (2, 4, and 6 wk,* P*<0.01). However, its content was evidently downregulated by XJEK or Fosinopril treatment for 2, 4, and 6 wk, compared with MI groups. As shown in Figure 3(a), the NT-ProBNP content was 770.81±59.22 pg·ml^−1^ versus 515.65±48.57 pg·ml^−1^ in 2 wk (*P*<0.01), 801.39±56.35 pg·ml^−1^ versus 569.98±51.88 pg·ml^−1^ in 4 wk (*P*<0.01), and 799.78±32.06 pg·ml^−1^ versus 563.31±17.14 pg·ml^−1^ in 6 wk (*P*<0.001) for MI versus XJEK treatments.

Compared with sham group at the same age, NT-ProBNP content in cardiac tissues was significantly higher in the rats with MI (2, 4, and 6 wk,* P*<0.05). However, XJEK and Fosinopril treatments could normalize the NT-ProBNP content (Figure 3(b)).

### 3.5. Effects of XJEK on Cardiac Hypertrophy and Cardiac Collagen Deposition in MI Rats

**Figure 4:**
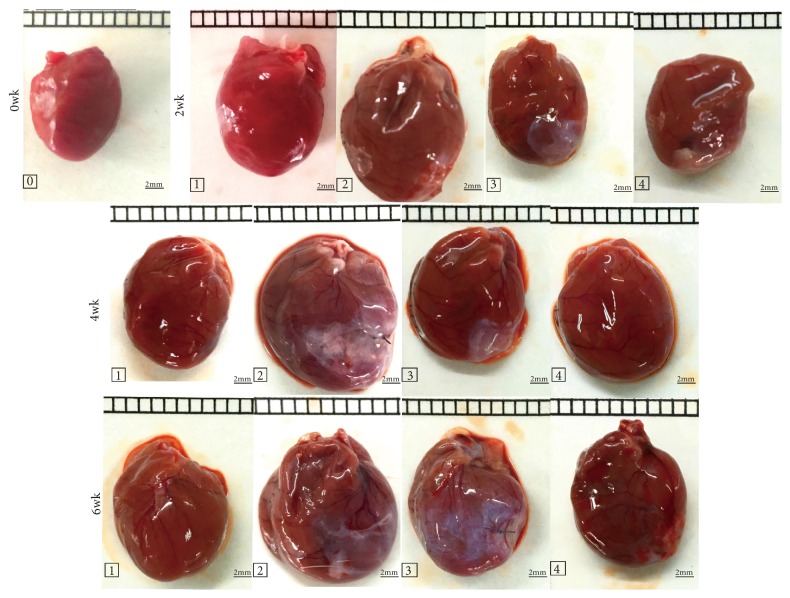
*Effects of XJEK on HW/BW in MI rats*. (0) Control group; (1) sham group; (2) MI group; (3) MI+XJEK group; (4) MI+Fosinopril group.

**Figure 5:**
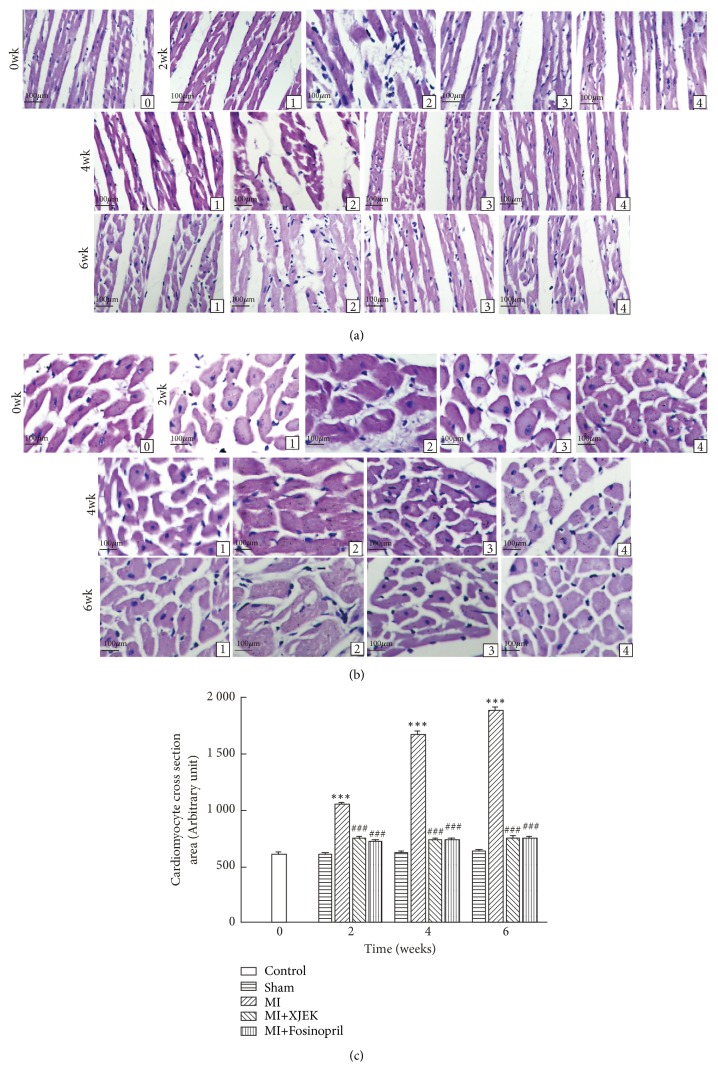
*Effects of XJEK on cardiomyocyte CSA, long axis in MI rats*. (HE stain, magnification×200). (a) Representative images of histological section of cardiomyocyte long axis; (b) representative images of histological section of cardiomyocyte cross-section; (c) Quantitative analyses results. (0) Control group; (1) sham group; (2) MI group; (3) MI+XJEK group; (4) MI+Fosinopril group. Data are represented as mean ±SEM (*n*=8~10). ^*∗∗∗*^*P*<0.001 versus sham group and ^*###*^*P*<0.001 versus MI group.

Morphological hypertrophy of heart was featured by apparently increased HW/BW ratio in MI groups (2 wk and 4 wk,* P*<0.01; 6 wk,* P*<0.05; Table 3 and Figure 4). Similarly, as indicated by hematoxylin-eosin (HE) staining of cardiac tissues from MI groups for 2, 4, and 6 wk, myocyte CSA, and longitudinal diameter increased markedly compared to those of sham groups (2, 4, and 6 wk,* P*<0.01; Figures 5(a), 5(b), and 5(c)). However, both the elevated HW/BW and myocyte CSA could be restored by XJEK or Fosinopril treatments for 2, 4, and 6 wk (2, 4, and 6 wk,* P*<0.01 or* P*<0.05).

**Figure 6:**
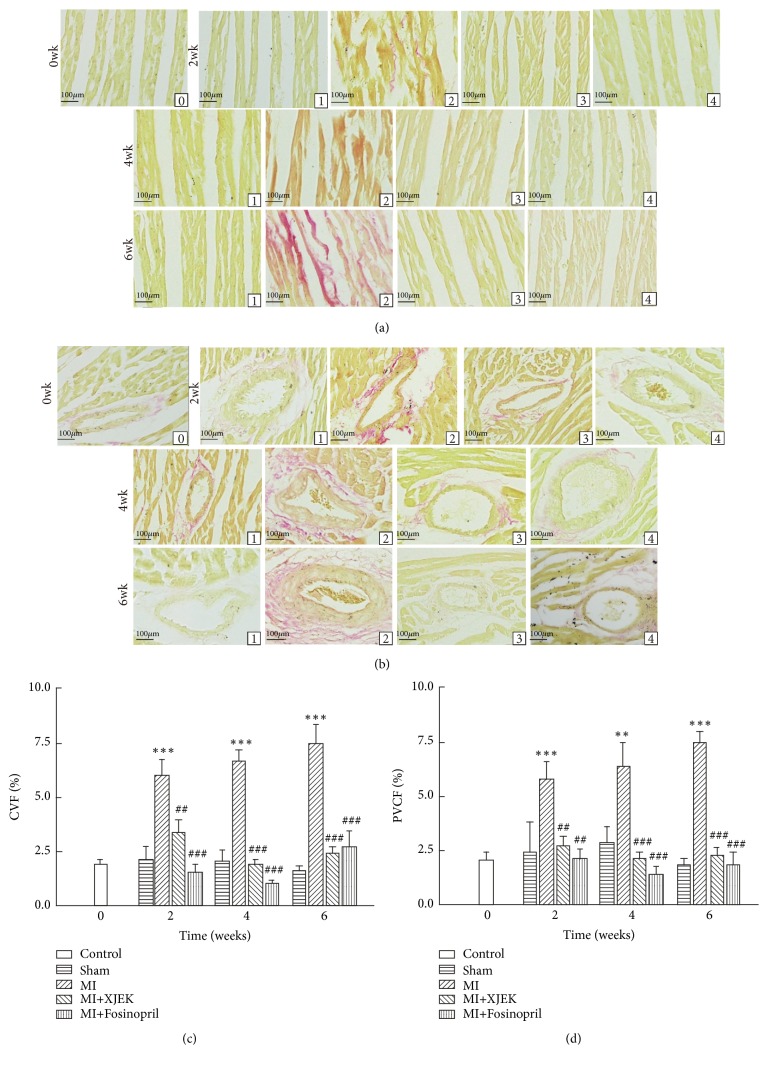
*Effects of XJEK on cardiac tissue CVF and PVCA in MI rats*. (a) Representative images of histological section of CVF; (b) Representative images of histological section of PVCA. ((c) and (d)) Quantitative analyses results. (0) Control group; (1) sham group; (2) MI group; (3) MI+XJEK group; (4) MI+Fosinopril group. Data are represented as mean ±SEM (*n*=8~10). ^*∗∗*^*P*<0.01 and ^*∗∗∗*^*P* <0.001 versus sham group, ^*##*^*P*<0.01 and ^*###*^*P*<0.001 versus MI group.

The effects of XJEK treatment for 2, 4, and 6 wk on CVF and PVCA in rat hearts were examined by VG staining. Compared with sham groups, the CVF (2, 4, and 6 wk* P*<0.01; Figures 6(a) and 6(c)) and PVCA (2, 4, and 6 wk* P*<0.01; Figures 6(b) and 6(d)) were significantly elevated in MI groups for 2, 4, and 6 wk, but evidently reduced in XJEK (2, 4, and 6wk,* P*<0.01) and Fosinopril (2, 4, and 6 wk,* P*<0.01) treatments for 2, 4, and 6 wk.

### 3.6. Effects of XJEK on Aortic Remodeling in MI Rats

**Figure 7:**
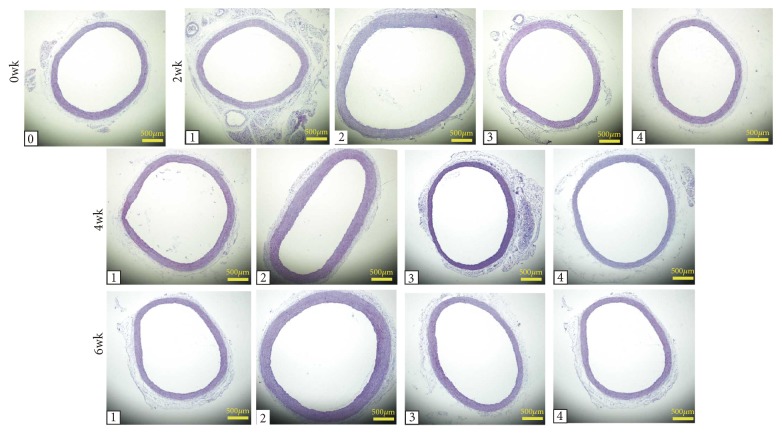
*Effects of XJEK on thoracic aorta remodeling in MI rats*. (HE stain, magnification ×40). (0) Control group; (1) sham group; (2) MI group; (3) MI+XJEK group; (4) MI+Fosinopril group.

The effects of XJEK treatment for 2, 4, and 6 wk on the vascular remodeling of the upper thoracic aorta was detected, respectively. Compared with sham group, MI groups for 2, 4, and 6 wk had an increasing trend on TAA, CSA, AR, and Media of the aorta (2, 4, and 6 wk,* P*<0.05 or* P*<0.01; Figure 7 and Table 4), while XJEK treatment for 2, 4, and 6 wk, the TAA, CSA, AR, and Media significantly decreased (2 wk, 4 wk, and 6wk,* P*<0.05 or* P*<0.01; Figure 6 and Table 3). Fosinopril treatment for 2, 4, and 6 wk achieved similar effects (2, 4, and 6 wk,* P*<0.05 or* P*<0.01; Figure 7 and Table 3).

### 3.7. Effects of XJEK on BH_4_, NO, and ADMA Content in MI Rats

**Figure 8:**
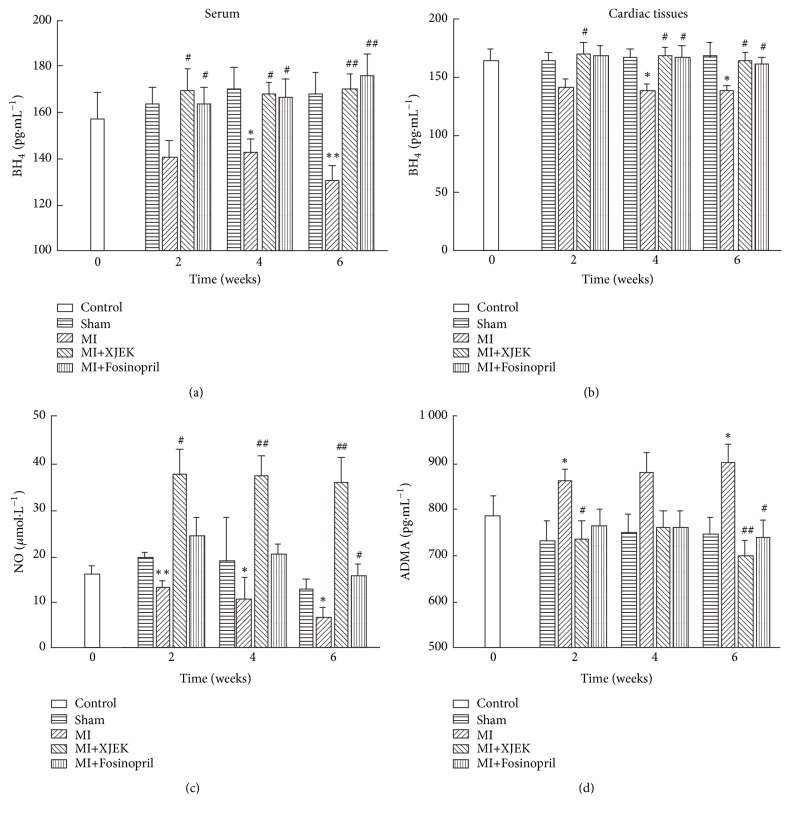
*Effects of XJEK on BH*_*4*_ *, NO and ADMA content in serum of MI rats*. (a) BH_4_ content in serum; (b) BH_4_ content in cardiac tissues; (c) NO content in serum; (d) ADMA content in serum. Data are represented as mean ±SEM (*n*=8~10). ^*∗*^*P*<0.05 and ^*∗∗*^*P*<0.01 versus sham group, ^*#*^*P*<0.05, and ^*##*^*P*<0.01 versus MI group.

BH_4_ content in serum and cardiac tissues of rats in MI groups for 2, 4, and 6 wk was examined by ELISA. BH_4_ was highly expressed in serum and cardiac tissues of rats in sham groups, while its content decreased in MI groups for 2, 4, and 6 wk (serum, 4 wk,* P*<0.05, and 6 wk,* P*<0.01, Figure 8(a); cardiac tissues, 4wk and 6wk,* P*<0.05, Figure 8(b)). However, its content was evidently upregulated by XJEK or Fosinopril treatment for 2, 4, and 6 wk, compared with MI groups.

NO content in serum of MI groups for 2, 4, and 6 wk was significantly decreased and deteriorated over time, compared with sham groups (2 wk,* P*<0.01, 4 wk and 6 wk,* P*<0.05). But both XJEK and Fosinopril treatment for 2, 4, and 6 wk could prevent NO reduction (Figure 8(c)).

We further examined the influence of XJEK treatment on ADMA levels in serum by ELISA. Compared with sham groups, serum ADMA levels tended to rise in MI groups for 2, 4, and 6 wk (4 wk and 6 wk,* P*<0.05.), while evidently descended in XJEK (2 wk,* P*<0.05 and 6 wk,* P*<0.01) and Fosinopril (6wk,* P*<0.05) treatment groups for 2, 4, and 6 wk (Figure 8(d)).

### 3.8. Effects of XJEK on ET-1 and Ang II Content in Serum and Cardiac Tissues of MI Rats

**Figure 9:**
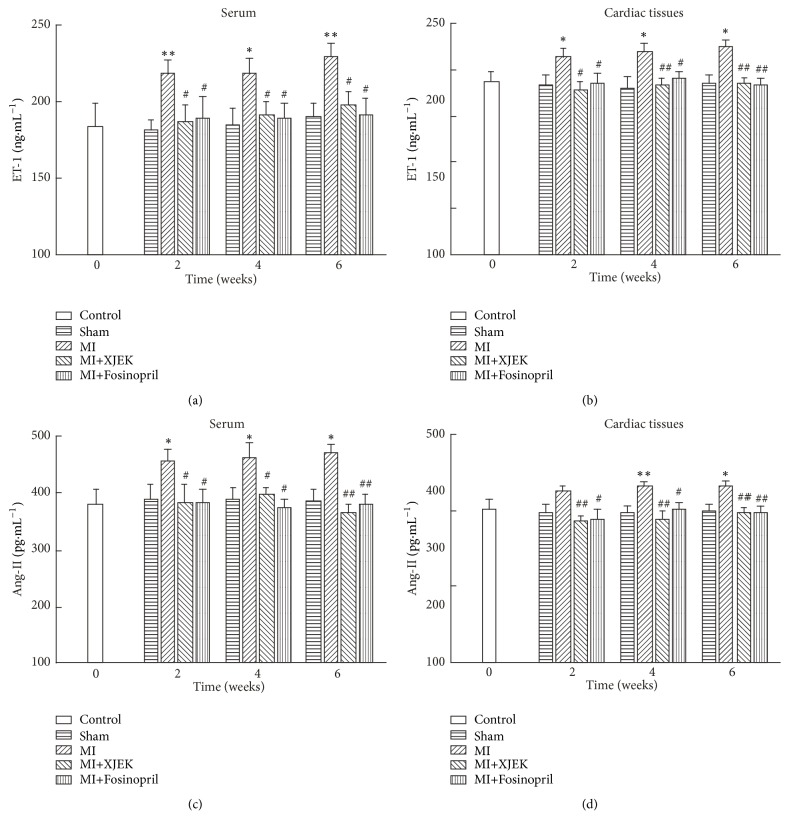
*Effects of XJEK on ET-1, and Ang II content in serum and cardiac of MI rats*. (a) ET-1 levels in serum; (b) ET-1 levels in cardiac tissues; (c) Ang II levels in serum; (d) Ang II levels in cardiac tissues. Data are represented as mean ± SEM (*n*=8~10). ^*∗*^*P*<0.05 and ^*∗∗*^*P*<0.01 versus sham group, ^*#*^*P*<0.05, ^*##*^*P*<0.01, and ^*###*^*P*<0.001 versus MI group.

ET-1 levels in serum and cardiac tissues of rats in MI groups for 2, 4, and 6 wk were examined by ELISA. Compared with the corresponding sham groups, the ET-1 levels in serum (2 wk, 6 wk* P*<0.01 and 4 wk* P*<0.05) and cardiac tissues (2, 4, and 6 wk* P*<0.05) were upregulated continuously in MI groups for 2, 4, and 6 wk (Figures 9(a) and 9(b)). However, the levels of ET-1 were significantly downregulated by XJEK or Fosinopril treatment for 2, 4, and 6wk (Figures 9(a) and 9(b)).

Similarly, Ang II levels in serum and cardiac tissues of rats in MI groups for 2, 4, and 6wk were determined. In comparison with sham groups, Ang II levels in serum (2, 4, and 6wk* P*<0.05) and cardiac tissues (4wk* P*<0.01 and 6wk* P*<0.05) increased evidently over time (Figures 9(c) and 9(d)). XJEK or Fosinopril treatment groups for 2, 4, and 6wk resulted in a marked decline in serum and cardiac tissue Ang II levels compared with MI groups (Figures 9(c) and 9(d)).

### 3.9. Effects of XJEK on ET_A_ Content in Cardiac Tissues of MI Rats

**Figure 10:**
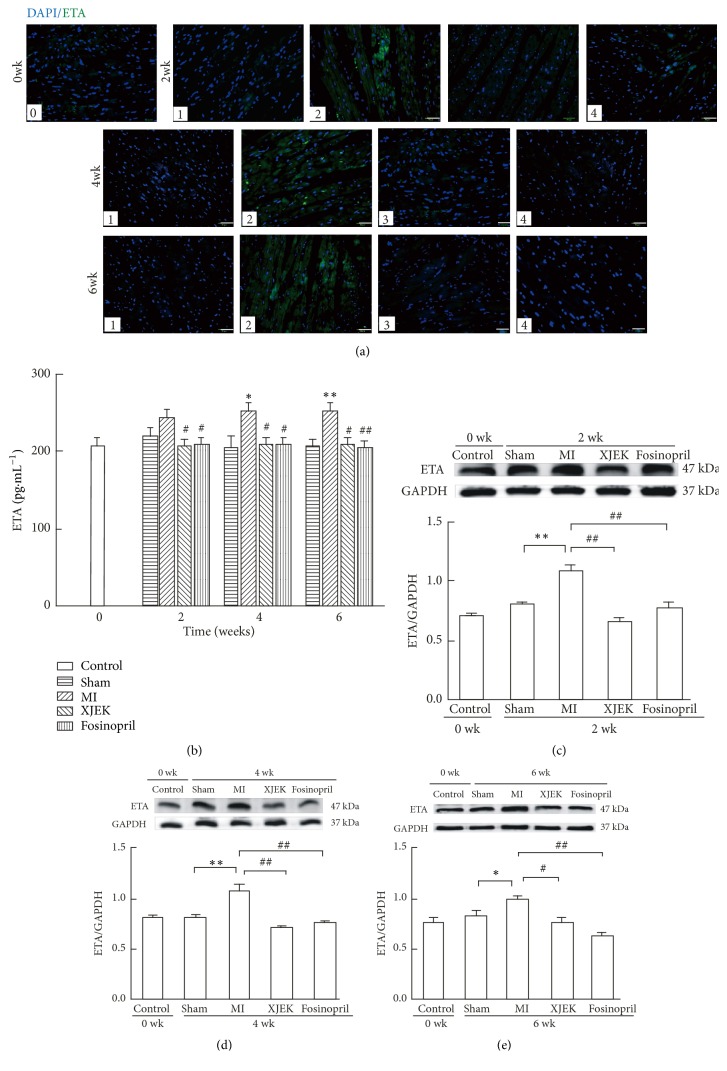
*Effects of XJEK on ET*_*A*_ *expression in MI rats*. (a) Representative images of Immunofluorescence of ETA (green) and DAPI (blue). (Scar bars: 20*μ*m); (b) ET_A_ levels in cardiac tissues by ELISA; ((c), (d) and (e)) ET_A_ content in cardiac tissues of rats for 2, 4 and 6wk by Western blot, respectively (mean±SEM,* n*=3). (0) Control group; (1) sham group; (2) MI group; (3) MI+XJEK group; (4) MI+Fosinopril group. Data are represented as mean ±SEM (*n*=8~10). ^*∗*^*P*<0.05 and ^*∗∗*^*P*<0.01 versus sham group, ^*#*^*P*<0.05 and ^*##*^*P*<0.01 versus MI group.

The expression of ET_A_ in cardiac tissues was examined by immunofluorescence staining, ELISA, and Western blot. There was virtually few stainings in sham groups, but in MI groups for 2, 4, and 6wk, intense green fluorescence indicated a higher expression level of ET_A_ compared with sham groups. XJEK or Fosinopril treatment for 2, 4, and 6wk obviously inhibited the increased ET_A_ levels (Figure 10(a)).

As shown in Figure 10(b) by ELISA detection, ET_A_ protein expression in cardiac tissues of MI rats was significantly higher than that in sham groups (4wk,* P*<0.05 and 6wk,* P*<0.01), but was evidently reduced in XJEK (2, 4 and 6wk,* P*<0.05) or Fosinopril (2wk and 4wk,* P*<0.05, 6wk,* P*<0.01) treatment for 2, 4, and 6wk (Figure 10(b)).

Consistently, the ET_A_ content in cardiac tissues of MI rats also had an obvious tendency to increase (2wk and 4wk,* P*<0.01, 6wk,* P*<0.05), compared with sham rats. However, both XJEK (2wk and 4wk,* P*<0.01, 6wk,* P*<0.05) and Fosinopril (2, 4, and 6wk,* P*<0.05) treatment could suppress the ET_A_ expression significantly (Figures 10(c), 10(d), and 10(e)).

### 3.10. Effects of XJEK on eNOS Content in Serum and Cardiac Tissues of MI Rats

**Figure 11:**
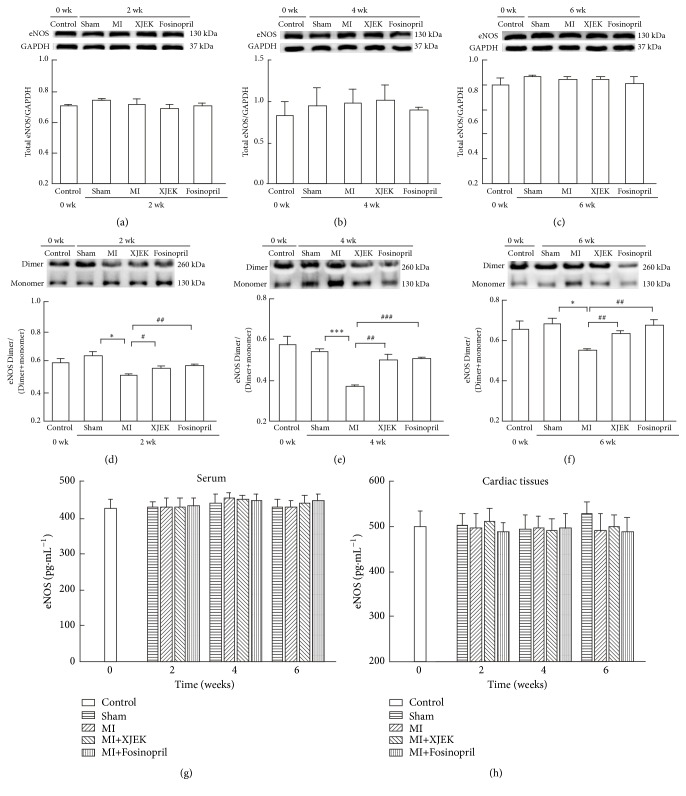
*Effects of XJEK on eNOS expression in serum and cardiac tissues of MI rats*. ((a), (b) and (c)) Representative Western blots of total eNOS expression in cardiac tissues and quantitative analyses of total eNOS standardized to GAPDH, data are represented as mean ±SEM (*n*=3); ((d), (e) and (f)) the same protein samples as in (a), (b), or (c) were subjected to low-temperature SDS-PAGE to assess eNOS dimers and monomers in cardiac tissues; ratio of eNOS dimer/ (dimer +monomer), data are represented as mean ±SEM (*n*=3); (g) eNOS expression in serum by ELISA, data are represented as mean ±SEM (*n*=8~10); (h) eNOS expression in cardiac tissues by ELISA, data are represented as mean ±SEM (*n*=8~10). ^*∗*^*P*<0.05 and ^*∗∗∗*^*P*<0.001 versus sham group, ^*#*^*P*<0.05, ^*##*^*P*<0.01, and ^*###*^*P*<0.001 versus MI group.

The expression of eNOS in the serum and cardiac tissues was examined by ELISA. The result showed that there were no significant differences in eNOS expression among all the experimental groups (Figures 11(g) and 11(h)).

To provide insights into the mechanisms underlying the observed protective effects of XJEK treatment on endothelial function, we probed into the effect of XJEK on the expression of total eNOS and eNOS dimer/ (dimer+monomer) ratio in cardiac tissues by Western bolt. There was no significant difference in total eNOS expression among sham, MI, XJEK, and Fosinopril treatment for 2, 4, and 6wk. On the other hand, the eNOS dimer/ (dimer+monomer) ratio was apparently lower in cardiac tissues of MI rats for 2, 4 and 6wk (2wk and 6wk,* P*<0.05, 4wk,* P*<0.01). But XJEK treatment significantly increased the dimer/ (dimer+monomer) ratio of eNOS protein expression (2wk,* P*<0.05, 4wk and 6wk,* P*<0.01). Fosinopril also markedly ameliorated the reduced eNOS dimer/ (dimer+monomer) ratio similarly to that by XJEK treatment (2, 4, and 6wk,* P*<0.01; Figures 11(d), 11(e), and 11(f)).

Altogether, these findings have indicated that MI is associated with the progression of eNOS uncoupling, but not total eNOS expression, in cardiac tissues of MI rats. XJEK could inhibit the eNOS uncoupling and thereby protecting endothelial function.

## 4. Discussion

In the present study, we found that XJEK significantly (1) suppressed the cardiovascular remodeling and ECG remodeling and improved cardiac function abnormalities in a rat model of MI for 2, 4 and 6wk; (2) alleviated the increasing levels of ET-1, ET_A_ and Ang II of each time point; (3) inhibited the reduction of NO, BH_4_ content and eNOS dimer/ (dimer+monomer) ratio therefore ameliorated ED. These results indicate that XJEK exerts a continuously beneficial effect on MI induced by LAD ligation in rats, which may be at least in part due to the restoration of cardiovascular structures, cardiac function, and especially endothelial function.

The very surgical ligation of animal LAD could simulate the clinical situation of MI, which has been extensively used for the pathophysiological study of post-MI CR [4, 25]. Being related to alterations in geometry, size and molecular phenotype, and MI-derived CR potentiates the development of ventricular arrhythmias, left ventricle (LV) dilatation and subsequent cardiac function abnormalities [26]. Of note, excessive myocardial fibrosis in MI rats contributes to diastolic and eventually systolic dysfunction by increasing myocardial stiffness and reducing pumping capacity [27]. Herein, cardiac function in this model becomes complicated due to its association with the process of infarct expansion, cardiac hypertrophy and ventricular remodeling. Moreover, LV hypertrophy and fibrosis post-MI may result in ECG remodeling which involves prolonged action potential duration and abnormal heart beats [28]. According to a recent study, cardiac arrhythmia disruption is potential risk factor for cardiac ischemia, sudden cardiac death, and stroke [29]. In line with the previous studies, the results of our analysis showed marked CR as reflected by elevated HW/BW ratio and CSA, more collagen deposition, prolonged QT interval duration, elevated ST segment, and widened P, T, and QRS waves post-MI (2, 4 and 6wk) which aggravated over time. The ST segment elevation is the most sensitive marker for myocardial infarction, and it reflects myocardial necrosis and the consequent loss of cell membrane in an injuring myocardium. Interestingly, XJEK reduces QRS, QT interval, P wave and T wave width, indicating that XJEK exerts protective effects on ECG remodeling. The T wave represents the repolarization time, and a prolonged T wave may be due to a delay in recovery and the depleted energy level in the ischemic tissue. Normalization of the ST segment, P, T, and QRS waves by XJEK indicates sufficient perfusion all the way through the myocardial microvasculature. As far as cardiac function is concerned, impaired LVSP, ±dp/dt_max_ and increased NT-ProBNP levels, which is recognized as a biomarker of heart failure [30], develop over time after MI. At the end of 2 and 4 weeks of the experiment, the rats with myocardial infarction were in a high hemodynamic state due to compensatory effects, while MI rats at 6wk were at the stage of transition from compensatory to decompensate and would even change into heart failure. However, the deteriorated CR was inhibited and the progressive cardiac function abnormalities were restored to normal condition after treatment with XJEK for 2, 4, and 6 wk.

The widely distributed vascular endothelium plays a very important role in modulating vascular tension by producing and releasing multiple endothelium-derived relaxing factors, including NO, prostacyclin, and endothelium-derived hyperpolarizing factors [31]. The evaluation of endothelial function in patients has gained increasing attention in the clinical settings due to its role as an excellent surrogate marker of cardiovascular events. Caused by the loss of NO and excessive vasoconstriction factors, ED has been shown to be associated with increased occurrence of MI and constitutes one of the earliest prognostic markers of cardiovascular disease [32]. Previous studies demonstrate that adequate level of endothelial NO is important for preserving normal vascular physiology, whereas decreased bioavailability of NO has been proposed as one of the central factors common for vascular remodeling, hypertension, and atherosclerosis [33]. eNOS is the key enzyme for NO synthesis which is expressed in the vascular endothelium widely. In common circumstance, functional eNOS works as a dimeric protein (coupled) together with the redox-sensitive cofactor BH_4_ and the substrate* L*-Arginine, to produce NO. In the presence of a highly oxidizing environment, exogenous BH_4_ is oxidized to BH_2_, which lacks of eNOS cofactor activity. A previous study suggested that the deletion of BH_4_ has been linked to eNOS uncoupling, and supplementation of BH_4_ is generally able to restore eNOS-mediated NO formation [34]. Uncoupled eNOS in dysfunctional endothelium generates O_2_^−^ and ONOO^–^, resulting in oxidation of BH_4_. Insufficient BH_4_, in turn, causes further eNOS uncoupling. Thus a vicious feedback loop is formed, which increases oxidative stress and reduces NO bioavailability [35]. ADMA is a structural analogue of* L*-Arginine, which can inhibit eNOS activation and competitively inhibit the production of endogenous NO, leading to ED in experimental chronic myocardial injury [36]. ET-1, a potent vasoconstrictor and proinflammatory peptide released mostly by vascular endothelial cells, is the predominant isoform expressed in vasculature and is the most potent vasoconstrictor currently known [37]. To the best of our knowledge, ET-1 binds to ET_A_ receptors em, leading to mitogenic reactions in cardiovascular cells ET-1 and NO interplay seems to have a great relevance in the physiological regulation of vascular tone and blood pressure. The imbalance between ET-1 and NO systems may be responsible for the pathogenesis of ED following cardiac hypertrophy [38]. Ang II is another biologically active peptide of RAS. High levels of Ang II, a biologically active hormone, could increase ET-1 expression in endothelial cells [38] and promote collagen deposition [39], thereby aggravating MI.

Consistent with the above statements, the present study showed that rats in MI groups for 2, 4, and 6wk displayed ED, elucidated by decreased NO and BH_4_ levels, and increased ADMA content in serum, as well as excessive concentration of potent vasoconstrictors such as Ang II and ET-1. In addition to these characteristics, MI rats in our study did not show an insufficiency in total eNOS levels. In contrast, a significant decrease was observed in the eNOS dimer/ (dimer+monomer) ratio in cardiac tissues of MI rats (Figures 11(a)–11(h)), which could be partially explained by the depletion of BH_4_ and increased ADMA. Taken together, both increased ADMA levels and the deficiency of BH_4_ could impair eNOS activity, resulting in eNOS monomerization and blocking the production of NO and simultaneously aggravating MI. Interestingly, our observations revealed that these abnormal changes of endothelial-related factors were alleviated by XJEK treatment, pointing to the ED-protective properties of XJEK. However, this study is restricted to* in vivo* situations and did not clearly dissect the cellular and molecular mechanism of Xin-Ji-Er-Kang-induced cardioprotection. Therefore, further studies should be performed* in vivo* and* in vitro* to elucidate the beneficial effects of XJEK on myocardial infarction.

## 5. Conclusion

The present study confirms that XJEK continuously protects against MI induced cardiac injury by reversing CR and cardiac function abnormalities over time. More importantly, it highlights the key role of XJEK in inhibiting ED through attenuation of eNOS uncoupling following MI. Our results suggest that XJEK may be a candidate for the development of a new therapeutic drug in the treatment of MI.

## Figures and Tables

**Table 1:** Effects of XJEK on ECG remodeling in MI rats.

Group	Time	Height of P(mv)	Width of P(ms)	Width of T(ms)	Time of Q-T(ms)	Time of P-R(ms)	Height of S(mv)
Control	0wk	0.10±0.01	19.50±1.53	27.67±0.85	52.67±1.62	46.33±1.53	-0.33±0.05

Sham	2wk	0.11±0.01	20.22±0.62	27.56±1.47	50.33±1.33	45.33±1.22	-0.23±0.02
MI		0.10±0.01^*∗∗*^	20.88±0.93	39.25±1.50^*∗∗∗*^	60.88±1.44^*∗∗∗*^	53.00±2.85^*∗*^	-0.15±0.03^*∗*^
MI+XJEK		0.12±0.01^#^	17.25±0.80	21.13±2.58^#^	40.63±3.76	45.43±1.54^#^	-0.25±0.03^#^
MI+Fosinopril		0.12±0.02^##^	18.88±2.17^#^	20.63±8.73^###^	20.14±2.41^##^	48.38±5.63^#^	-0.25±0.08^#^

Sham	4wk	0.11±0.01	17.45±1.06	29.22±1.61	53.56±1.97	44.45±1.47	-0.43±0.07
MI		0.08±0.01^*∗*^	26.40±1.45^*∗∗∗*^	37.38±3.96	60.22±3.70	55.40±0.82^*∗∗∗*^	-0.16±0.03^*∗∗*^
MI+XJEK		0.13±0.01^##^	18.09±0.97^##^	22.30±2.37^#^	51.00±3.54	45.73±2.14^##^	-0.34±0.06^##^
MI+Fosinopril		0.10±0.01	19.18±1.13^##^	26.50±2.85^#^	53.20±2.51	51.78±1.53^#^	-0.28±0.02^##^

Sham	6wk	0.10±0.01	15.90±1.18	27.89±1.21	48.44±1.32	47.44±1.25	-0.36±0.04
MI		0.07±0.01^*∗*^	22.44±1.17^*∗∗*^	38.50±2.82^*∗∗*^	59.63±3.30^*∗∗*^	51.22±2.47	-0.15±0.04^*∗∗*^
MI+XJEK		0.10±0.01^#^	19.33±0.72^#^	29.50±2.69^#^	51.60±3.34	45.10±1.62^#^	-0.31±0.04^#^
MI+Fosinopril		0.10±0.01^##^	18.55±0.65^##^	27.45±2.71^#^	50.40±2.46^#^	45. 20±1.31^#^	-0.30±0.04^#^

Data are represented as mean±SEM (*n*=8~10). ^*∗*^*P*<0.05, ^*∗∗*^*P*<0.01, and ^*∗∗∗*^*P* <0.001versus sham group; ^*#*^*P*<0.05, ^*##*^*P*<0.01, and ^*###*^*P*<0.001 versus MI group.

**Table 2:** Effects of XJEK on cardiac function injury in MI rats.

Group	Time	HR (Times/min)	ASBP (mmHg)	LVEDP (mmHg)	LVSP (mmHg)	+dp/dtmax (mmHg/s)	-dp/dtmax (mmHg/s)
Control	0wk	381.63±33.58	96.67±2.92	-16.42±3.88	109.51±2.83	4049.67±237.23	-3317.31±183.80

Sham	2wk	403.14±38.88	95.31±3.76	-23.47±3.79	119.82±3.06	3972.84±122.19	-3546.74±220.85
MI		450.00±32.07	109.80±4.06^*∗*^	-8.92±4.57^*∗*^	126.38±4.20	4572.79±227.81^*∗*^	-4031.96±183.64
MI+XJEK		376.86±42.73	92.97±3.94^##^	-15.67±8.09	109.07±4.78^#^	3687.44±228.53^#^	-3302.12±262.12^#^
MI+Fosinopril		400.38±23.00	93.83±3.86^#^	-14.69±2.89	102.89±5.34^##^	3246.49±198.24^###^	-3012.45±329.80^#^

Sham	4wk	372.64±12.31	91.64±1.64	-27.43±1.79	113.58±3.46	3749.03±212.07	-3390.87±201.06
MI		428.00±50.08	110.92±4.63^*∗∗*^	-9.21±4.25^*∗∗∗*^	135.18±4.73^*∗∗*^	4541.46±308.14^*∗*^	-4292.16±757.17^*∗*^
MI+XJEK		399.50±18.09	90.34±2.57^###^	-20.65±1.90	107.83±3.67^##^	3402.62±130.43^##^	-2983.99±139.16^##^
MI+Fosinopril		386.40±41.21	92.41±2.85^##^	-20.53±1.37^#^	106.28±11.90^##^	3402.17±235.64^##^	-3305.10±383.84^##^

Sham	6wk	387.30±18.66	103.51±4.83	-28.18±2.69	119.76±7.11	4279.16±402.39	-3694.09±289.27
MI		402.50±17.21	90.91±4.48	-7.26±7.39	107.51±6.02	3445.35±416.49	-2870.24±244.74^*∗∗*^
MI+XJEK		389.33±10.04	113.42±4.32^##^	-22.98±1.98^#^	129.06±4.97^#^	4876.77±369.09^#^	-4270.13±210.55^##^
MI+Fosinopril		385.82±28.82	115.23±2.72^##^	-23.84±1.77^#^	128.82±11.65^##^	4937.62±203.45^##^	-4117.43±228.77^##^

Data are represented as mean±SEM (*n*=8~10). ^*∗*^*P*<0.05, ^*∗∗*^*P*<0.01, and ^*∗∗∗*^*P* <0.001versus sham group; ^*#*^*P*<0.05, ^*##*^*P*<0.01, and ^*###*^*P*<0.001 versus MI group.

**Table 3:** Effects of XJEK on BW, HW, and HW/BW in MI rats.

Group	Time	BW(g)	HW(g)	HW/BW
Control	0wk	268.5±4.31	0.78±0.03	2.92±0.13

Sham	2wk	339.29±7.74	0.97±0.05	2.77±0.09
MI		330.40±9.84	1.07±0.05	3.24±0.11^*∗∗*^
MI+XJEK		337.38±7.49	0.99±0.04	2.96±0.06^#^
MI+Fosinopril		328.50±4.41	0.95±0.02^#^	2.94±0.07^#^

Sham	4wk	392.00±12.50	1.01±0.03	2.61±0.11
MI		374.67±13.07	1.07±0.05	2.85±0.07^*∗∗*^
MI+XJEK		380.25±8.11	0.98±0.03^#^	2.58±0.10^#^
MI+Fosinopril		377.00±9.94	0.97±0.02^#^	2.56±0.06^##^

Sham	6wk	419.78±17.96	0.99±0.03	2.47±0.11
MI		396.86±16.29	1.11±0.05^*∗*^	2.79±0.04^*∗*^
MI+XJEK		407.67±9.41	1.00±0.02^#^	2.57±0.07^#^
MI+Fosinopril		412.45±10.60	0.98±0.02^##^	2.38±0.06^##^

BW: body weight; HW: heart weight; HW/BW: heart weight index. Data are represented as mean±SEM (*n*=8~10). ^*∗*^*P*<0.05 and ^*∗∗*^*P*<0.01 versus sham group; ^*#*^*P*<0.05 and ^*##*^*P*<0.01 versus MI group.

**Table 4:** Effects of XJEK on thoracic aorta remodeling in MI rats.

Group	Time	TAA(10^3^um^2^)	LA(10^3^um^2^)	CSA(10^3^um^2^)	AR(um)	LR(um)	MT(um)
Control	0wk	376.57±11.65	285.52±10.55	91.06±4.23	345.97±5.40	301.15±5.62	44.81±2.01

Sham	2wk	396.45±14.85	308.04±13.86	88.41±7.81	354.78±6.73	312.56±7.11	42.23±1.27
MI		460.73±24.59^*∗*^	334.60±17.95	126.13±7.64^*∗∗*^	381.95±10.47^*∗*^	325.49±8.99	56.47±2.21^*∗∗*^
MI+XJEK		424.75±20.05^#^	323.49±17.03^#^	101.26±4.21^###^	366.73±8.70^#^	319.84±8.45	46.89±1.33^###^
MI+Fosinopril		358.72±11.05^##^	267.88±8.64^#^	90.84±3.28^##^	337.68±5.13^##^	291.78±4.65^#^	45.89±1.23^##^

Sham	4wk	428.24±16.97	327.46±14.02	100.78±3.23	368.68±7.45	322.32±7.04	46.36±0.72
MI		467.81±24.91	332.99±16.64	134.82±8.66^*∗∗*^	384.77±9.80	324.71±7.86	60.05±2.24^*∗∗∗*^
MI+XJEK		348.88±19.14^##^	257.17±18.13^##^	91.71±1.80^###^	331.76±9.48^###^	283.98±10.52^#^	47.78±1.28^###^
MI+Fosinopril		415.35±17.90	311.45±16.26	103.90±4.08^##^	362.73±7.99	313.69±8.59	49.04±1.99^##^

Sham	6wk	447.41±13.95	333.27±16.07	113.43±4.43	376.25±5.61	323.73±7.51	51.81±2.56
MI		570.04±41.35^*∗*^	421.32±24.98^*∗*^	148.72±17.33	423.50±15.27^*∗*^	364.79±10.72^*∗*^	58.70±5.08
MI+XJEK		457.76±17.77	341.10±19.27	116.66±4.35^#^	381.28±7.44	328.70±9.42	52.58±2.71^#^
MI+Fosinopril		409.38±23.97^#^	303.47±19.51^#^	105.91±6.26^##^	359.23±10.63^#^	308.87±10.37	50.37±2.30^###^

TAA: area of total aorta; LA: area of lumen; CSA: cross-sectional area; AR: aorta radius; LR: luminal radius; MT: media thickness. (mean±SEM, *n*=8~10). Data are represented as mean±SEM (*n*=8~10). ^*∗*^*P*<0.05, ^*∗∗*^*P*<0.01, and ^*∗∗∗*^*P* <0.001versus sham group, ^*#*^*P*<0.05, ^*##*^*P*<0.01, and ^*###*^*P*<0.001 versus MI group.

## Data Availability

The data used to support the findings of this study are available from the corresponding author upon request.

## Abbreviations

XJEK: Xin-Ji-Er-Kang
MI: Myocardial infarction
CR: Cardiovascular remodeling
ED: Endothelial dysfunction
TTC: Triphenyltetrazolium chloride
NO: Nitric oxide
ELISA: Enzyme-linked immunosorbent assay
ECG: Electrocardiogram
NT-ProBNP: N-terminal probrain natriuretic peptide
ET-1: Endothelin-1
ET_A_: Endothelin receptor A
Ang II: Angiotensin II
ADMA: Asymmetric dimethylarginine
BH_4_: Tetrahydrobiopterin
eNOS: Endothelial NO synthase
ASBP: Artery systolic blood pressure
LVSP: Left ventricular systolic
LVEDP: Left ventricular end-diastolic pressures
±dp/dtmax: Rate of rise of left ventricular pressure
RAS: Renin-angiotensin system
HE: Hematoxylin and eosin
VG: Van Gieson
CSA: Cross-sectional area
TAA: Total aorta area
AR: Aorta radius
MT: Media thickness
PVCA: Perivascular collagen area
CVF: Collagen volume fraction.
